# Source-Dependent Proteomic and Fatty-Acid Responses of Yak, Holstein Cow, Donkey and Bactrian Camel Colostrum to Short-Time Heat Treatment

**DOI:** 10.3390/foods15203609

**Published:** 2026-10-10

**Authors:** Jie Zhao, Jingjing Wang, Qian Liu, Jiaying Zhuang, Chong Chen, Yiquan Zhang

**Affiliations:** 1 College of Agriculture and Animal Husbandry, Qinghai University, Xining 810016, China; 15202531350@163.com (J.Z.); 18992506514@163.com (J.W.); 17397018983@163.com (Q.L.); 13256987471@163.com (J.Z.); 2 Department of Nutrition and Health, China Agricultural University, No. 17 Qinghua East Road, Haidian District, Beijing 100083, China; chenchong409@cau.edu.cn

**Keywords:** early postpartum milk, source-associated variation, thermal stability, bioactive components, functional dairy products

## Abstract

Colostrum undergoes rapid compositional changes during the early postpartum period, and standardized sampling is essential for cross-species comparison. In this study, day-3 postpartum milk from yak, Holstein cow, donkey, and Bactrian camel (*n* = 3 per source), representing an early transitional stage between colostrum and mature milk was analyzed using DIA proteomics and targeted GC-MS fatty-acid profiling. Samples were divided into paired unheated and heat-treated aliquots (72 °C for 15 s). Clear source-dependent differences were observed in both the proteomic and FAME-derived fatty-acid profiles, and these differences remained largely preserved after heating. Heat treatment affected the proteome more extensively than the fatty-acid profile, with 387, 146, 303, and 276 differential proteins identified in yak, Holstein cow, donkey, and Bactrian camel milk, respectively, compared with 14, 6, 0, and 0 differential FAME features. Source × heat-treatment analysis further identified 48 protein features and eight FAME features whose responses differed among the four milk sources. Six protein symbols were shared across all four heat-responsive protein sets, whereas the FAME response was mainly concentrated in yak and Holstein cow milk. Cross-omic integration showed strong agreement between proteomic and fatty-acid profiles at the animal-source level (Procrustes *M*^2^ = 0.276, *p* < 0.001; Mantel *r* = 0.943, *p* = 0.001), whereas heat-induced changes were more omics-specific. Overall, animal source was the primary factor associated with the molecular composition of early postpartum milk, whereas short-time heating introduced smaller but clearly source-dependent alterations, particularly at the proteomic level. These source-associated molecular characteristics provide insights into the functional diversity of early lactation milk and support the identification of bioactive components and the development of value-added dairy products.

## 1. Introduction

Colostrum is the first mammary secretion produced after parturition and plays an essential role in supporting neonatal growth and immune development. Compared with mature milk, colostrum contains higher concentrations of immunoglobulins, whey proteins, growth factors, and other bioactive components, which contribute to passive immunity transfer, antimicrobial defense, and nutritional regulation [1,2]. Despite its biological value, colostrum remains poorly utilized in large-scale dairy production because of its unfavorable processing characteristics. The high abundance of heat-sensitive proteins, particularly immunoglobulins, may result in protein aggregation and coagulation during thermal processing, affecting processing stability and product quality [2,3]. In addition, differences in sensory properties and chemical composition between colostrum and mature milk may negatively influence the technological performance of conventional dairy products [2]. Therefore, understanding the molecular changes associated with processing is important for improving the potential utilization of colostrum as a valuable dairy resource.

Thermal treatment represents a major challenge for colostrum utilization because heating is required during dairy processing to ensure product safety. Regarding bovine colostrum, Elizondo-Salazar et al. reported that thermal processing altered immunoglobulin retention and reduced the concentration of heat-sensitive bioactive proteins, with the magnitude of these changes depending on temperature–time combinations [4]. Similarly, Gazi et al. demonstrated that heating affected the physicochemical properties and structural stability of bovine colostrum proteins, leading to changes in protein functionality under different processing conditions [5]. These findings indicate that thermal exposure does not affect all colostrum components equally. Therefore, identifying molecular components that remain stable or undergo changes during heating is essential for understanding processing adaptability and preserving the functional potential of colostrum.

Current knowledge regarding thermal responses of colostrum is mainly derived from bovine studies, whereas comparative information among different animal sources remains limited [4,6]. Milk from different species possesses distinct molecular architectures, but it remains unclear whether these compositional differences translate into different responses during thermal processing. Most previous investigations have focused on individual species or selected components, making it difficult to distinguish universal heat-induced changes from source-dependent responses. A systematic comparison of multiple animal-derived colostrum matrices under identical processing conditions is therefore required to understand the molecular basis of processing adaptability.

The four animal sources were selected to represent both a conventional dairy reference and compositionally distinct alternative dairy matrices. Holstein cow served as the conventional dairy reference. Yak represents a high-altitude dairy resource whose milk is typically richer in total solids, protein, and fat than conventional cow milk [7]. Donkey milk has a distinctive whey-protein profile and is particularly rich in lysozyme [8], whereas Bactrian camel milk contains naturally occurring heavy-chain antibodies in addition to a distinct whey-protein composition [9]. These contrasting characteristics made the four sources suitable for testing whether a common high-temperature short-time (HTST) challenge elicits broadly conserved or source-associated molecular responses.

The postpartum sampling stage is another critical factor in comparative colostrum research. Colostrum composition changes rapidly after parturition, particularly during the first several days, when immunoglobulins, whey proteins, and other bioactive components progressively decrease as milk transitions toward mature milk [10]. Therefore, the postpartum collection time should be clearly defined when comparing milk from different animal sources. In this study, samples were collected at day-3 postpartum, representing an early transitional stage between immediate postpartum colostrum and mature milk. Although milk secreted immediately after parturition contains the highest levels of immune-related components, it is primarily required for neonatal passive immunity acquisition and is often limited in availability, which restricts comparative studies involving multiple animal species and biological replicates [1,11]. Therefore, day-3 postpartum samples were selected to enable standardized sampling among different animal species while representing a defined early lactation stage. Although recent omics approaches have greatly expanded our understanding of milk composition, the relationship between interspecies molecular differences and thermal responses of colostrum remains largely unexplored. In particular, it is unknown whether molecular differences among animal sources mainly reflect baseline compositional variation or determine different processing behaviors after heating. Addressing this gap is important for evaluating the processing potential of diverse dairy resources and developing appropriate utilization strategies.

Therefore, this study compared early postpartum milk from yak, Holstein cow, donkey, and Bactrian camel using a paired experimental design. Samples were subjected to 72 °C for 15 s, corresponding to a widely applied HTST pasteurization condition in the dairy industry [12]. This condition was selected as a standardized thermal challenge to evaluate molecular responses under a defined heat exposure while avoiding excessive disruption of the milk matrix. DIA proteomics and targeted GC-MS fatty-acid profiling were integrated to address three questions: (i) whether animal origin or thermal processing is the dominant factor shaping molecular characteristics; (ii) whether identical heat treatment induces conserved or source-associated molecular responses; and (iii) which molecular features contribute to differences in processing adaptability among animal sources. We hypothesized that species-associated differences would represent the major source of baseline molecular variation in colostrum, whereas short-time heating would introduce source-dependent alterations in protein and fatty-acid components due to differences in intrinsic molecular characteristics. The findings provide new insights into the heat responsiveness of diverse dairy resources and support the rational utilization of colostrum-derived products.

## 2. Materials and Methods

### 2.1. Sample Collection and Experimental Design

Colostrum was collected in April 2025 on day-3 postpartum from yaks (*Bos grunniens*), Holstein cows (*Bos taurus*), donkeys (*Equus asinus*), and Bactrian camels (*Camelus bactrianus*). The animals were not calving on the same calendar day; however, all samples were collected strictly at the same postpartum stage (day 3 after parturition). Specifically, yak colostrum samples were collected on 3 April, 7 April, and 13 April; Holstein cow colostrum samples were collected on 5 April, 7 April, and 25 April; donkey colostrum samples were collected on 6 April, 16 April, and 18 April; and Bactrian camel colostrum samples were collected on 9 April, 22 April, and 24 April. Three individual animals were independently sampled from each source, and samples from different animals were not pooled, resulting in 12 biological samples (*n* = 3 per animal source). Three biological replicates per animal source were adopted for this exploratory paired-omics comparison. The paired aliquot design prioritized the within-animal comparison of heated and unheated samples and was not intended to capture the full population-level biological diversity of each species. Yak colostrum was collected in Tongren, Qinghai Province, China (35.248° N, 102.253° E; 3473 m above sea level); Holstein cow colostrum in Huangzhong District, Xining (36.568° N, 101.614° E; 2100 m); donkey colostrum in Huzhu Tu Autonomous County, Haidong (36.682° N, 102.027° E; 2350 m); and camel colostrum in Ulan County, Haixi Prefecture (36.998° N, 98.197° E; 3000 m). The four animal sources were obtained from commercial farms located in different regions of Qinghai Province, China. Animals were maintained under the conventional feeding and management practices of their respective farms. The yak and Bactrian camel herds were managed predominantly under extensive/semi-extensive grazing systems, whereas Holstein cows were maintained under an intensive housed feeding system and donkeys under a semi-intensive system. Because yak, Holstein cow, donkey, and Bactrian camel represent different production systems, feeding regimes were not unified across species. However, animals within each source were maintained under consistent farm conditions, and all samples were collected, stored, and processed using identical procedures. Therefore, comparisons among animal sources were interpreted as source-associated differences considering both biological characteristics and production conditions.

Basic physicochemical characterization was performed on the unheated samples before thermal treatment. Protein content was determined by the Kjeldahl method [13], and crude protein was calculated from total nitrogen using a conversion factor of 6.38. Fat content was determined by the Gerber method [14]. The pH was measured directly using a pHS-S3C pH meter (Shanghai Youke Instrument and Meter Co., Ltd., Shanghai, China). The resulting protein content, fat content, and pH values are summarized in Table 1.

Samples were stored at −80 °C until analysis. Before thermal treatment, frozen samples were transferred from the freezer to a 4 °C refrigerator and thawed overnight under refrigerated conditions. During thawing, samples were kept in sealed tubes to prevent contamination and moisture exchange. After complete thawing, samples were gently homogenized by inversion to ensure uniform distribution of components and immediately subjected to subsequent experimental procedures. Repeated freeze–thaw cycles were avoided throughout the experiment. Each biological sample was divided into two matched aliquots: one remained unheated and the other was heat-treated, generating 12 matched aliquot pairs and 24 analytical samples. A–D represented yak, Holstein cow, donkey, and camel, respectively; the first digit represented the treatment (1, heat-treated; 2, unheated), and the final digit represented the biological replicate. Thus, A11 and A21 were paired aliquots from the same yak. The study used routinely collected colostrum for compositional analysis and involved no experimental intervention or invasive procedures on the animals.

### 2.2. Heat Treatment

For heat treatment, 10 mL of colostrum was transferred to a 15 mL centrifuge tube and heated in an HH-6 water bath (Shanghai Bilon Instruments Co., Ltd., Shanghai, China). Sample temperature was monitored at the center of the liquid using an external calibrated digital temperature probe (model 1650, Yangzi, Chuzhou, Anhui, China). The temperature probe was calibrated before experiments using a certified temperature reference standard. The 15 s holding period began only when the sample temperature reached 72 °C, after which the tube was immediately transferred to an ice bath. The matched unheated aliquot was handled in parallel.

### 2.3. DIA Proteomic Analysis

#### 2.3.1. Protein Fraction Preparation and Tryptic Digestion

Colostrum samples were first centrifuged at 2000× *g* for 15 min at 4 °C using a refrigerated centrifuge (model LR-10M, Hunan Hexi Instrument & Equipment Co., Ltd., Changsha, Hunan, China) to remove the cream layer and reduce interference from milk fat globule membrane (MFGM)-associated components. The skimmed samples were subsequently centrifuged at 14,000× *g* for 30 min at 4 °C, and the resulting supernatant fraction was collected for DIA-based proteomic analysis. The identified proteins therefore represent the heat-responsive changes in the soluble protein fraction obtained after defatting and centrifugation. Protein concentration was determined by the bicinchoninic acid assay [15] and protein quality was assessed by SDS-PAGE [16]. Triethylammonium bicarbonate (TEAB), tris(2-carboxyethyl)phosphine (TCEP), iodoacetamide (IAA), acetone, and sequencing-grade trypsin were purchased from Beijing Solarbio Science & Technology Co., Ltd. (Beijing, China). Triethylammonium bicarbonate (TEAB), tris(2-carboxyethyl)phosphine (TCEP), iodoacetamide (IAA), acetone, and sequencing-grade trypsin were purchased from the same supplier.For each sample, 30–50 μg of protein was adjusted to 100 mmol/L triethylammonium bicarbonate, reduced with 10 mmol/L tris(2-carboxyethyl)phosphine at 37 °C for 60 min, and alkylated with 40 mmol/L iodoacetamide for 40 min at room temperature in the dark. Proteins were precipitated with 800 μL acetone at −20 °C for 2 h, collected by centrifugation (12,000× *g*, 15 min), washed with 800 μL of 90% acetone, air-dried for 5 min, and digested overnight at 37 °C with trypsin at an enzyme-to-protein ratio of 1:40 (*w*/*w*). Peptides were acidified to pH < 3, desalted, eluted with 70% acetonitrile, and quantified using a NanoDrop One spectrophotometer (Thermo Fisher Scientific, Waltham, MA, USA).

#### 2.3.2. LC-dia-PASEF Mass Spectrometry

Equal peptide amounts were analyzed using a Vanquish Neo UHPLC system (Thermo Fisher Scientific, USA) coupled to a timsTOF Ultra 2 trapped-ion-mobility mass spectrometer (Bruker Daltonics, Bremen, Germany). Peptides were separated on a home-packed 15 cm × 100 μm column (1.7 μm particles). Acetonitrile and formic acid were purchased from Xining Baiyabo Biotechnology Co., Ltd. (Xining, Qinghai, China). Mobile phase A was 2% acetonitrile/0.1% formic acid and mobile phase B was 80% acetonitrile/0.1% formic acid. The gradient was 8% B at 0 min, 17% B at 1 min, 55% B at 5.5 min, and 99% B at 7 min, followed by a hold at 99% B to 8 min. Data were acquired in positive-ion dia-PASEF mode [17], with a source voltage of 1.6 kV and an m/z range of 100–1700. The MS2 scheme used overlapping 10 m/z isolation windows spanning approximately m/z 343.6–1136.6, with corresponding trapped-ion-mobility centers of approximately 0.74–1.26.

#### 2.3.3. Protein Identification and Annotation

Raw dia-PASEF data were processed in Spectronaut 20.5.260227.92449 (Biognosys AG, Schlieren, Switzerland) using directDIA and BGS Factory Settings [18]. Samples from each animal source were searched against the corresponding species-specific UniProt FASTA database: *Bos grunniens* (Taxon ID 30521), *Bos taurus* (Taxon ID 9913), *Equus asinus* (Taxon ID 9793), and *Camelus bactrianus* (Taxon ID 9837). Peptide-spectrum match, peptide, and protein-group false discovery rates were controlled at 1%. Protein abundance was quantified from MS2 peak areas using automatic LFQ, cross-run normalization, and interference correction; no missing-value imputation was applied. PG.Quantity values were used in the cloud-analysis workflow. Protein annotations were assigned using UniProt [19], Gene Ontology [20], KEGG [21], eggNOG [22], and Pfam [23].

### 2.4. Targeted Fatty-Acid Profiling

#### 2.4.1. Standards and Sample Preparation

Fatty acids were analyzed after derivatization to fatty acid methyl esters (FAMEs) by targeted GC-MS [24]. A 51-component FAME mixture (CDAA-252893, ANPEL, Shanghai, China) was used for external calibration. n-Hexane, dichloromethane, methanol, and sodium hydroxide (NaOH) were purchased from Qinghai Laiyiner Biotechnology Co., Ltd. (Xining, Qinghai, China). A primary mixed standard was prepared by dissolving 100 mg of the FAME mixture in 1.0 mL n-hexane; an intermediate standard was prepared by diluting 100 μL of stock with 900 μL n-hexane, followed by serial preparation of eight calibration levels. For extraction, 100 μL colostrum was mixed with 1.0 mL dichloromethane/methanol (1:1, *v*/*v*), sonicated at 40 kHz for 15 min at 4 °C, held at −20 °C for 15 min, and centrifuged at 13,000× *g* for 10 min at 4 °C. A 500 μL aliquot of the lower organic phase was evaporated under nitrogen. Because quantification was based directly on FAME calibration, compound-level GC-MS results are reported using FAME names; the term “fatty-acid profile” refers to the corresponding biological fatty-acid composition represented by these derivatives.

The dried extract was mixed with 0.5 mL of 0.5 mol/L NaOH in methanol, vortexed for 30 s, sonicated at 40 kHz for 10 min at 4 °C, and incubated at 60 °C for 30 min. After cooling, 0.5 mL n-hexane was added; samples were vortexed and centrifuged (13,000× *g*, 10 min, 4 °C). A 100 μL aliquot of the upper phase was transferred to an autosampler vial. Samples above the calibration range were diluted tenfold.

#### 2.4.2. GC-MS Analysis and Quality Control

FAMEs were analyzed using an Agilent 8890 gas chromatograph coupled to an Agilent 7000D mass spectrometer (Agilent Technologies, Santa Clara, CA, USA) with a DB-FAST FAME capillary column (30 m × 0.25 mm i.d., 0.25 μm). Helium was used at 1.0 mL/min. The inlet was maintained at 250 °C; 1 μL was injected at 10:1 split. The oven program was 80 °C initially, 70 °C/min to 177 °C with a 2 min hold, then 5 °C/min to 230 °C with a 1 min hold. Electron ionization was performed at 70 eV in selected-ion-monitoring mode. Ion-source, quadrupole, and transfer-line temperatures were 250, 150, and 240 °C, respectively. Peaks were integrated using MassHunter Quantitative Analysis 10.0.707.0 with automatic integration followed by manual inspection. External calibration used analyte FAME peak area versus FAME standard concentration, and results were expressed as μg/mL of the corresponding FAME.

A 10 μg/mL mixed FAME standard was injected after every 5–10 study samples. Instrument-response RSDs for all 51 targeted FAMEs were below 15%. Thirty-eight FAMEs corresponding to 38 fatty acids were detected in study samples and retained for statistical analysis.

### 2.5. Statistical and Pathway Analysis

Proteomic and targeted FAME data were analyzed using the Majorbio cloud-analysis workflows supplied with the project reports. PCA was used to visualize the global sample structure; the proteomic dataset was also evaluated by sample-correlation analysis, whereas the FAME dataset was evaluated by hierarchical clustering. The predefined within-source treatment contrasts were A1 versus A2, B1 versus B2, C1 versus C2, and D1 versus D2 for yak, Holstein cow, donkey, and camel, respectively. Differential-protein numbers and higher/lower-abundance classifications were obtained from the platform output, and volcano plots were interpreted according to the same platform criteria.

Differential FAME features between groups were identified using a two-group comparison workflow. Statistical significance was defined as *p* < 0.05, and fold change (FC) was calculated as the ratio of mean abundance between the two groups to indicate the direction and magnitude of variation. FC values greater than 1 indicated increased abundance in the first group, whereas FC values lower than 1 indicated decreased abundance. Differential-protein KEGG enrichment analysis was performed with Benjamini–Hochberg correction for multiple testing [25]. Bubble plots displayed the rich factor on the *x*-axis and pathway terms on the *y*-axis, with bubble size representing the number of enriched proteins and color representing the adjusted *p*-values.

Direct source effects were quantified from the six pairwise comparisons among the four animal sources in the unheated state (A2–D2) and the heat-treated state (A1–D1). For proteomic interaction analysis, abundances sharing the same gene symbol were summed within each sample, and features with positive values in all 24 samples were retained. Each biological pair was expressed as log2(heat-treated/unheated); paired responses were compared among sources and corrected by the Benjamini–Hochberg procedure [25]. Platform-defined differential-protein lists were mapped to gene symbols for exact shared and source-specific response intersections.

The same paired-change framework was applied to FAME features with positive concentrations in all 24 samples. The platform criterion (*p* < 0.05; fold-change threshold = 1) was retained for within-source and cross-source differential sets, which were also summarized by exact intersections.

### 2.6. Integrative Proteome-Fatty-Acid Analysis

Cross-omic integration followed the supplied Majorbio association-analysis report. Overall configuration concordance was evaluated by Procrustes analysis and the Mantel test [26,27]. O2PLS was used to characterize joint and data-block-specific multivariate variation [28,29]. Protein–FAME associations were evaluated by Pearson correlation analysis and visualized as a clustered correlation heatmap. The platform also generated exploratory multiblock visualizations using the mixOmics package [30] in R (version 4.3.2).

Hierarchical integration used complete-case protein accessions (>0.1 in all 24 samples) and FAME features with positive concentrations in all samples. Log2-transformed, feature-standardized matrices were evaluated by Mantel analysis globally, after within-source centering, and for the 12 paired heat-treated-minus-unheated change vectors; permutations for the latter two analyses were restricted within the source. Source-level response magnitude was summarized as the RMS paired log2 response. Interaction-significant protein and FAME features were further integrated by Pearson correlation across paired responses, with a second analysis after within-source centering.

## 3. Results

### 3.1. Proteomic Profiles Differed Primarily Among Animal Sources

**Figure 1:**
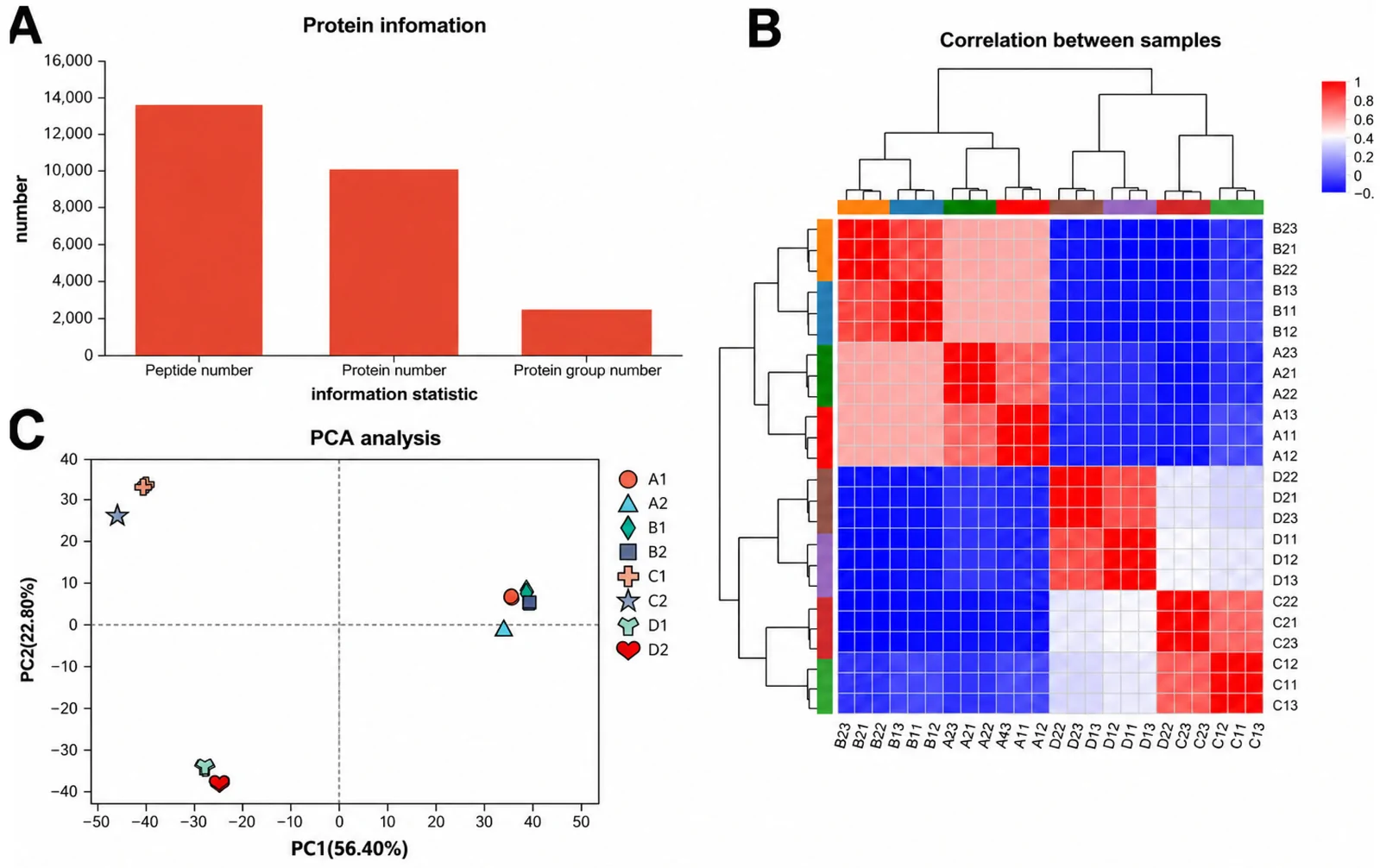
Global DIA proteomic profiles of yak, Holstein cow, donkey, and Bactrian camel colostrum before and after heat treatment. (**A**) Protein-identification summary. (**B**) Sample-correlation heatmap based on protein-abundance profiles; the red-to-blue color scale indicates higher-to-lower correlation coefficients, and the colored annotation bars distinguish the different sample groups. (**C**) PCA score plot of the 24 samples; PC1 and PC2 explained 56.4% and 22.8% of total proteomic variation, respectively.

DIA proteomics identified 13,642 peptides, 10,120 protein entries, and 2583 protein groups across the 24 samples (Figure 1A). The correlation matrix separated the samples into four groups corresponding to yak, Holstein cow, donkey, and Bactrian camel colostrum (Figure 1B). Heated and unheated aliquots from the same source remained close to one another, whereas correlations between different sources were lower. Yak and Holstein cow were the most similar pair. PCA gave the same result: PC1 and PC2 explained 56.4% and 22.8% of the variance, respectively, and the four sources were clearly separated, while heating caused only a small displacement within each source (Figure 1C).

### 3.2. Proteomic Differences Among Colostrum Sources Were Largely Maintained After Heating

The six pairwise comparisons among unheated samples contained 722, 1136, 1096, 1220, 1196, and 837 differential proteins for yak–Holstein, yak–donkey, yak–camel, Holstein–donkey, Holstein–camel, and donkey–camel, respectively. Yak and Holstein cow showed the fewest differential proteins, whereas the largest differences were observed for Holstein–donkey and Holstein–camel. After heating, the corresponding numbers were 733, 1046, 1071, 1143, 1148, and 858. The counts before and after heating were highly correlated (*r* = 0.991, *p* = 0.00013), showing that the relative proteomic differences among the four colostrum sources changed little after 72 °C/15 s treatment.

### 3.3. Proteomic Changes Induced by Short-Time Heating

**Figure 2:**
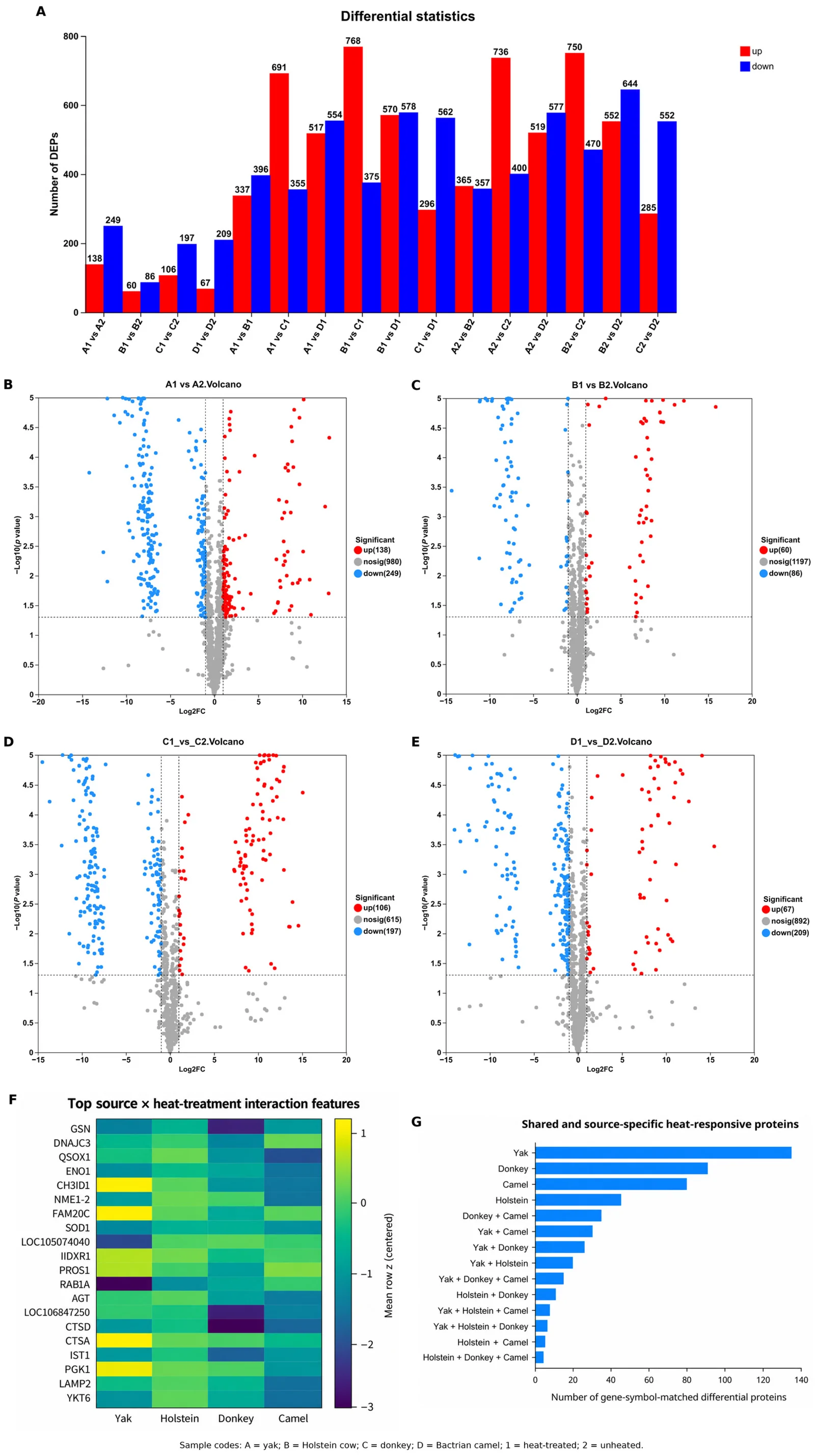
Treatment-associated proteomic differences and source-dependent interaction patterns. (**A**) Numbers of differential proteins among the predefined comparisons. (**B**–**E**) Volcano plots for yak (A1 vs. A2), Holstein cow (B1 vs. B2), donkey (C1 vs. C2), and Bactrian camel (D1 vs. D2), respectively. Red and blue points indicate proteins with significantly higher and lower abundance, respectively, whereas gray points indicate non-significant proteins. The gray dashed vertical lines indicate the fold-change threshold, and the horizontal dashed line indicates the significance threshold (*p* < 0.05). (**F**) Mean paired log2(heat-treated/unheated) responses of the top 20 gene-symbol-harmonized protein features with significant source × heat-treatment interaction. (**G**) Exact intersections of gene-symbol-matched heat-responsive proteins among the four animal sources.

The proteomic response to 72 °C/15 s heating differed substantially among the four colostrum sources (Figure 2A–E). Yak showed the largest number of differential proteins (387), followed by donkey (303) and Bactrian camel (276), whereas only 146 proteins were significantly altered in Holstein cow. In every source, proteins with lower abundance after heating outnumbered those with higher abundance, but the imbalance differed considerably. The proportion of decreased proteins was 64.3% in yak, 58.9% in Holstein cow, 65.0% in donkey, and 75.7% in camel. Camel therefore showed the clearest shift toward lower protein abundance, while Holstein cow had the smallest differential set and the least pronounced directional imbalance.

### 3.4. Most Heat-Responsive Proteins Were Specific to Individual Colostrum Sources

Paired interaction analysis identified 48 protein features whose responses to heating differed significantly among the four colostrum sources (FDR < 0.05; Figure 2F). Two response patterns were evident among the leading interaction proteins. Some proteins retained the same direction of change but differed considerably in magnitude. GSN decreased in all four sources, with the strongest reduction in donkey, whereas PROS1 and SOD1 increased across the four sources but to different extents. Other proteins changed direction among sources. NME1-2 showed a strong positive response in yak but a negative response in camel; DDR1 decreased markedly in yak but increased in the other three sources; and CTSD was positive in yak and Holstein cow but negative in donkey and camel. CTSA showed an especially strong decrease in donkey, while QSOX1 shifted from positive responses in yak and Holstein cow to negative responses in donkey and camel. These patterns indicate that the interaction was produced by both differences in response magnitude and genuine reversals in the direction of the heat response.

The intersection analysis further showed that most heat-responsive proteins were source-specific (Figure 2G). Gene-symbol-matched source-specific sets contained 137 proteins in yak, 91 in donkey, 82 in camel, and 43 in Holstein cow. Only six proteins—ASL, C8B, FGB, FGG, RPLP2, and TPP1—were identified as heat-responsive across all four colostrum sources. Among these shared proteins, C8B and FGG showed consistent decreases after heating, whereas FGB exhibited source-dependent directional changes.

### 3.5. Functional Profiles of Heat-Responsive Proteins Differed Among Colostrum Sources

**Figure 3:**
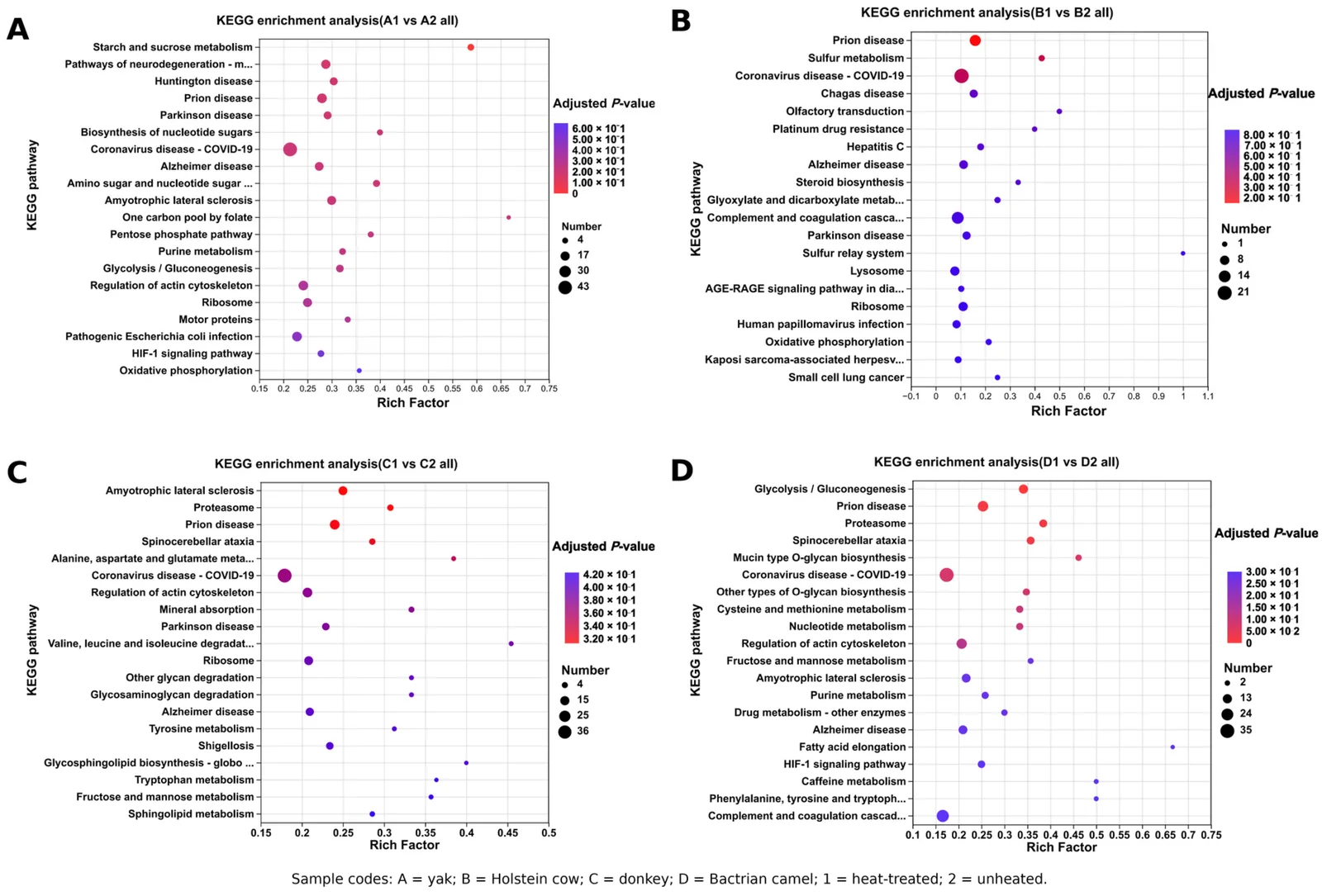
KEGG enrichment of treatment-responsive proteins in yak (**A**), Holstein cow (**B**), donkey (**C**), and Bactrian camel (**D**) colostrum. Bubble position represents rich factor, bubble size represents the number of annotated proteins, and color represents the Benjamini–Hochberg-adjusted *p* value.

KEGG enrichment analysis revealed distinct functional annotation profiles among the heat-responsive protein sets from different colostrum sources (Figure 3). In yak colostrum, carbohydrate metabolism-related pathways were among the enriched pathways. Starch and sucrose metabolism showed the highest enrichment among the identified pathways, with a high rich factor and an adjusted *p*-value of 0.011 (Figure 3A). Other enriched pathways included biosynthesis of nucleotide sugars, amino sugar and nucleotide sugar metabolism, glycolysis/gluconeogenesis, the pentose phosphate pathway, and one-carbon metabolism.

Donkey showed a different functional profile despite having a similarly large number of differential proteins. Proteasome, alanine/aspartate/glutamate metabolism, branched-chain amino-acid degradation, regulation of the actin cytoskeleton, mineral absorption, glycan degradation, and sphingolipid metabolism were among the leading terms (Figure 3C). Holstein cow, which had the smallest differential-protein set, showed a more dispersed distribution involving sulfur metabolism, steroid biosynthesis, glyoxylate and dicarboxylate metabolism, complement and coagulation cascades, lysosome, and other categories (Figure 3B).

Bactrian camel showed a distinct KEGG enrichment pattern (Figure 3D). Glycolysis/gluconeogenesis and proteasome were among the enriched pathways, together with mucin-type O-glycan biosynthesis, cysteine and methionine metabolism, nucleotide metabolism, and regulation of the actin cytoskeleton. Complement and coagulation cascades also contained a relatively large number of heat-responsive proteins, including C8B, FGB, and FGG. Among these shared proteins, C8B and FGG showed consistent decreases after heating, whereas FGB displayed source-dependent directional changes (Figure 2G).

Yak and donkey exhibited different KEGG enrichment patterns despite both showing relatively large numbers of heat-responsive proteins. Carbohydrate metabolism-related pathways were more frequently enriched in yak, whereas proteasome-, amino acid metabolism-, and glycan-related pathways were more prominent in donkey.

### 3.6. Fatty-Acid Profiles Differed Markedly Among Colostrum Sources

**Figure 4:**
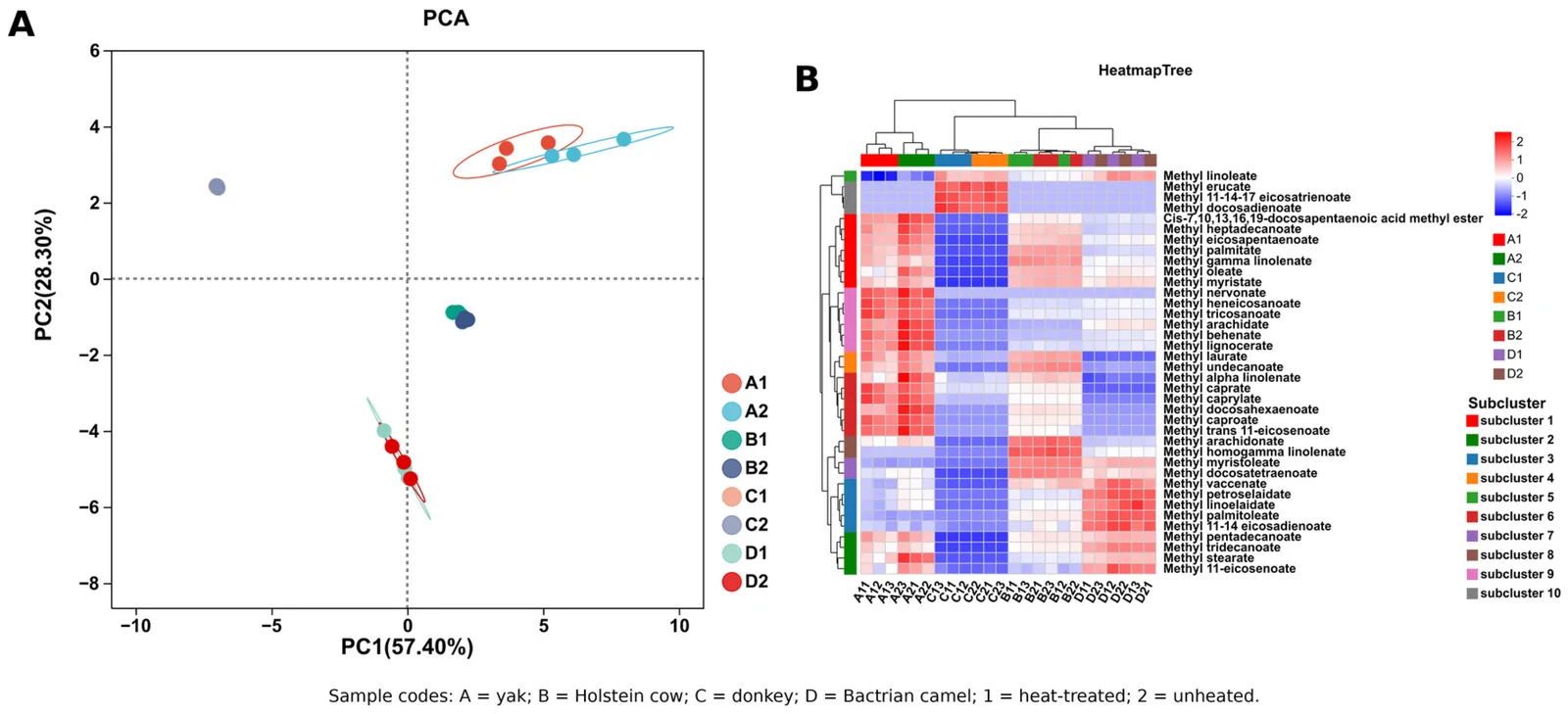
Global targeted FAME-derived fatty-acid profiles of the four colostrum sources. (**A**) PCA score plot; PC1 and PC2 explained 57.4% and 28.3% of total variation, respectively. (**B**) Hierarchical clustering heatmap showing standardized abundance patterns of the 38 quantified FAMEs across the 24 samples.

Targeted GC-MS quantified 38 FAMEs across the four colostrum sources. PCA showed a clear separation among yak, Holstein cow, donkey, and Bactrian camel colostrum, with PC1 and PC2 accounting for 57.4% and 28.3% of the total variance, respectively (Figure 4A). Donkey colostrum was particularly distinct along PC1, whereas yak, Holstein cow, and camel occupied separate regions on the opposite side of the score plot. In contrast, heated and unheated samples from the same source remained close to each other, indicating that the differences associated with the animal source were much greater than those produced by the 72 °C/15 s treatment.

The heatmap further showed that the separation was produced by different combinations of FAMEs rather than by a uniform increase or decrease in total fatty acids (Figure 4B). Donkey colostrum showed relatively high standardized abundances of methyl linoleate, methyl erucate, methyl 11,14,17-eicosatrienoate, and methyl docosadienoate, together with lower abundances of many saturated and monounsaturated FAMEs. Yak colostrum, in contrast, showed relatively high abundances of methyl palmitate, methyl oleate, methyl myristate, and several longer-chain saturated FAMEs. Camel colostrum was characterized by higher abundances of several FAMEs in the lower part of the heatmap, including methyl vaccenate, methyl petroselaidate, methyl linolelaidate, and methyl palmitoleate. These source-specific combinations account for the strong separation observed in the PCA.

The pairwise comparisons were consistent with the PCA and heatmap. Before heating, all 38 quantified FAMEs met the differential criterion in the yak–donkey comparison, whereas 37 were differential between Holstein cow and donkey and 28 between yak and Holstein cow. After heating, the extent of these source-level differences was largely maintained, with 37, 37, and 24 differential FAMEs identified in the corresponding comparisons, respectively. Across all six source pairs, the numbers of differential FAMEs before and after heating were strongly correlated (r = 0.971, *p* = 0.00123), indicating that the relative extent of FAME-profile differences among animal sources was largely preserved after heating.

### 3.7. Heat Treatment Altered Selected FAMEs Mainly in Yak Colostrum

**Figure 5:**
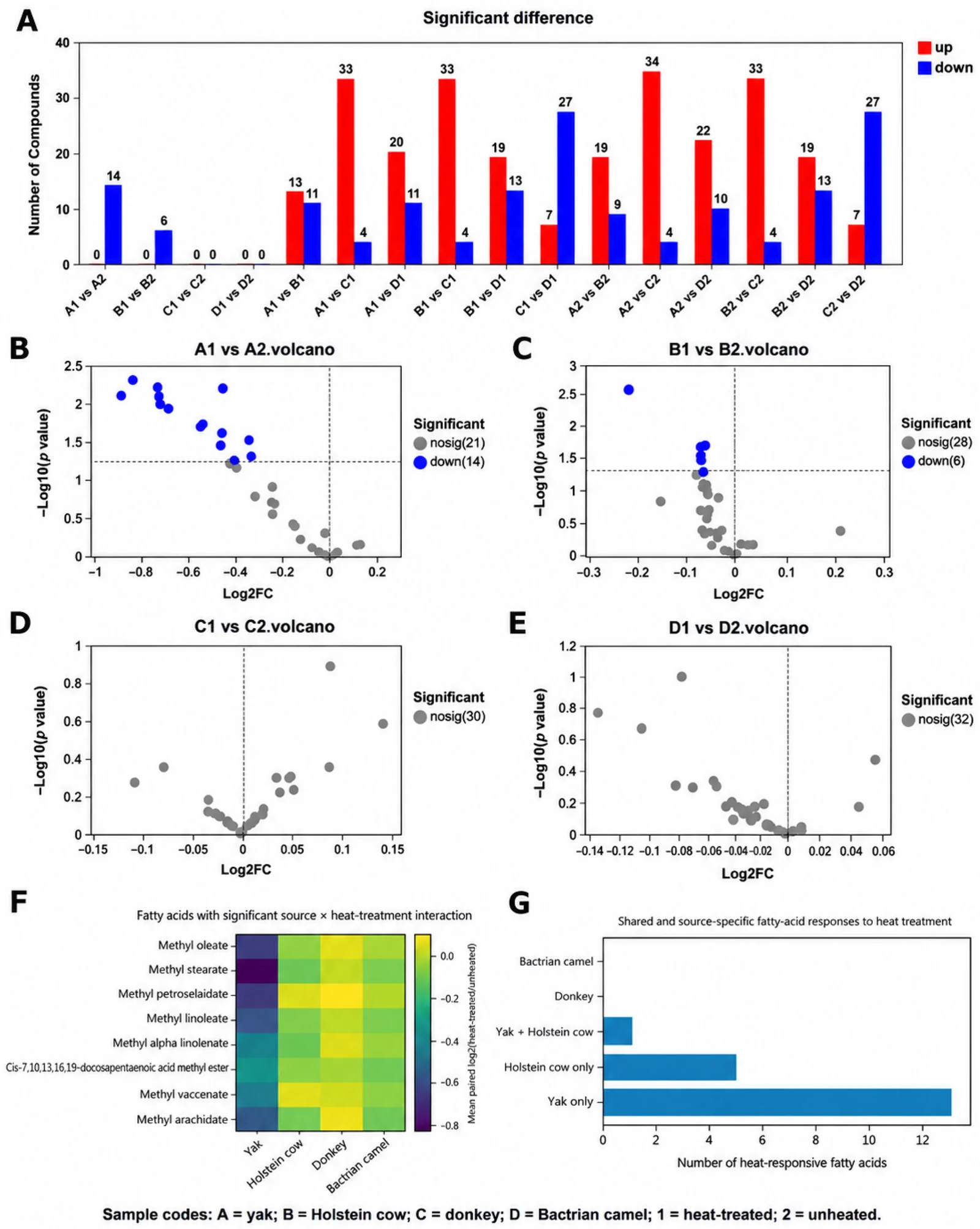
Targeted FAME-derived fatty-acid responses to heat treatment and source-dependent interaction patterns. (**A**) Numbers of higher- and lower-abundance differential FAME features among the predefined comparisons. (**B**–**E**) Volcano plots for yak, Holstein cow, donkey, and Bactrian camel, respectively. (**F**) Mean paired log2(heat-treated/unheated) responses of the eight FAME features with significant source × heat-treatment interaction. (**G**) Exact intersections of the four within-source heat-responsive FAME sets.

Pairwise comparisons revealed clear differences between animal-source effects and heat-treatment effects on FAME profiles (Figure 5A). Comparisons among animal sources contained 24–38 differential FAMEs, whereas within-source comparisons after heat treatment identified 14 heat-responsive FAMEs in yak and six in Holstein cow; no significant heat-responsive FAMEs were detected in donkey or Bactrian camel. All 14 heat-responsive FAMEs identified in yak and all six identified in Holstein cow showed decreased relative abundance after heating. Volcano plot analysis further showed that significant features were predominantly located in the negative log2(FC) region in yak and Holstein cow, with a stronger reduction pattern observed in yak (Figure 5B–E).

Comparison of heat-induced changes among the four sources further identified eight FAMEs showing source-dependent heat responses (Figure 5F): methyl oleate, methyl stearate, methyl petroselaidate, methyl linoleate, methyl α-linolenate, methyl docosapentaenoate, methyl vaccenate, and methyl arachidate. All eight FAMEs showed the largest negative log2(heat-treated/unheated) changes in yak among the four sources examined. The differences were particularly evident for methyl oleate, methyl stearate, and methyl linoleate, with mean log2(heat-treated/unheated) values of −0.718, −0.841, and −0.674 in yak, respectively, whereas corresponding changes in Holstein cow, donkey, and Bactrian camel were smaller. The differential FAMEs included both unsaturated fatty acids (oleate, linoleate, α-linolenate, vaccenate, and docosapentaenoate) and saturated fatty acids (stearate and arachidate).

The intersection analysis also showed that the FAME changes in yak and Holstein cow involved largely different compounds (Figure 5G). Thirteen FAMEs were unique to yak and five to Holstein cow, with methyl α-linolenate as the only shared differential FAME. Thus, the stronger response in yak was accompanied by a broader set of affected fatty acids, rather than simply a larger change in the same FAMEs that responded in Holstein cow.

### 3.8. Cross-Omic Integration Revealed Different Responses of Proteins and FAMEs to Heating

**Figure 6:**
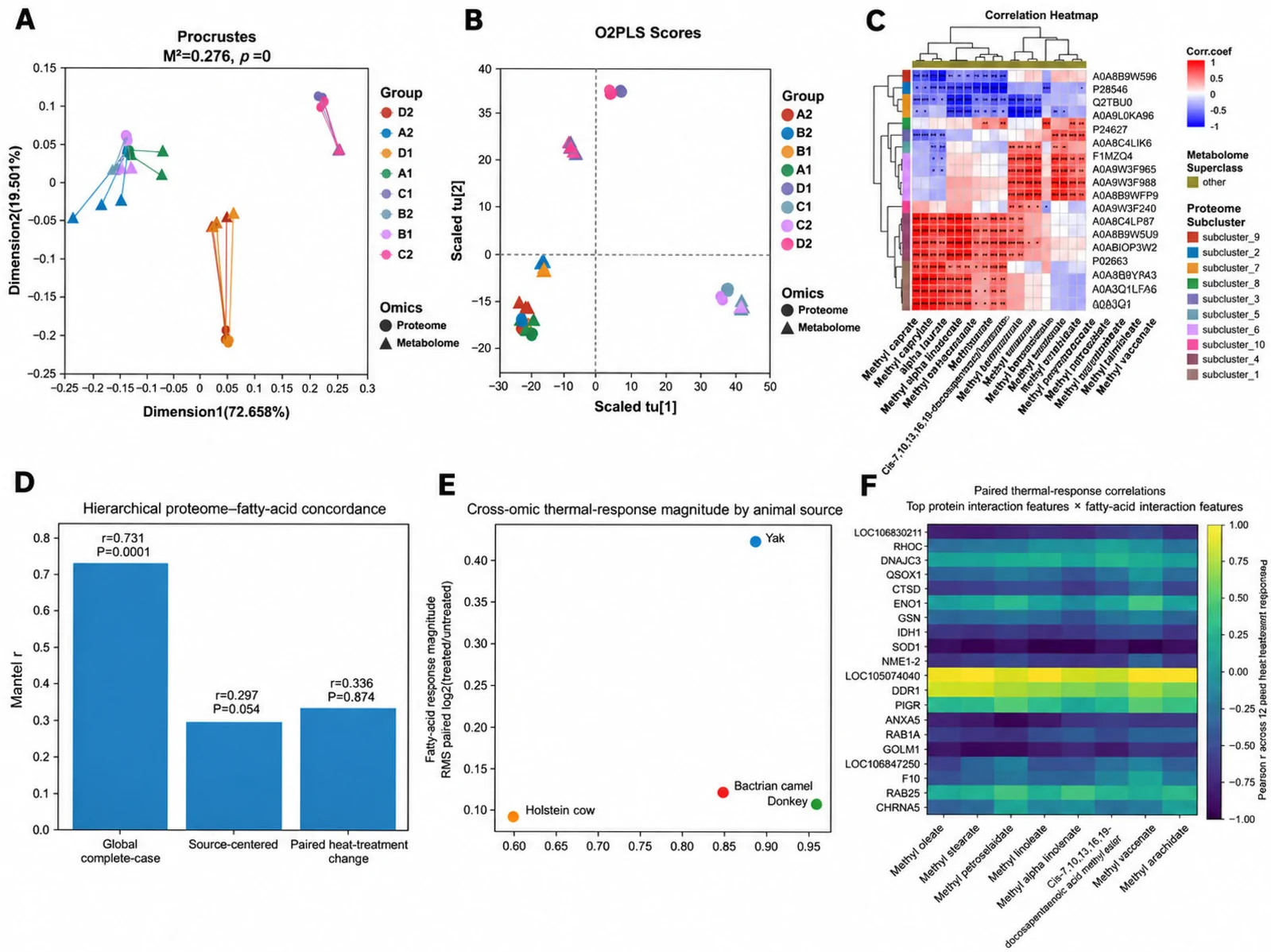
Integrated proteome−FAME analysis across animal sources and paired heat-treatment responses. (**A**) Procrustes analysis comparing the sample configurations derived from the proteomic and FAME datasets. (**B**) O2PLS score plot showing the joint structure of the proteomic and FAME data blocks. (**C**) Clustered Spearman correlation heatmap between selected protein accessions and quantified FAMEs. Red and blue indicate positive and negative correlations, respectively. Asterisks indicate the statistical significance of the correlations: *p* < 0.05, *p* < 0.01, and *p* < 0.001. (**D**) Hierarchical Mantel analysis based on global complete-case data, source-centered data, and paired heat-treatment change vectors. (**E**) Cross-omic heat-treatment response magnitude, summarized as the root mean square (RMS) of paired log2(heat-treated/unheated) values for each animal source. (**F**) Pearson correlation heatmap between the top protein interaction features and the eight FAME interaction features across the 12 paired heat-treatment responses.

The proteomic and FAME datasets showed strong overall agreement in sample-level variation across the 24 samples. Procrustes analysis showed significant concordance between the two datasets (*M*^2^ = 0.276, *p* < 0.001), and the Mantel test also indicated a strong correlation between the corresponding distance matrices (*r* = 0.943, *p* = 0.001) (Figure 6A). In both datasets, samples were separated primarily according to animal species, whereas heated and unheated samples from the same species remained relatively close. O2PLS showed the same pattern, with most of the modeled variation in both blocks assigned to the joint component (*R*^2^Xjoint = 0.6953; *R*^2^Yjoint = 0.9171; Figure 6B). Thus, the major variation shared by the two omic layers corresponded to differences among yak, Holstein cow, donkey, and Bactrian camel colostrum, whereas short-time heating introduced comparatively smaller shifts within each animal group.

The protein–FAME correlation heatmap also contained clear positive and negative modules (Figure 6C). Proteins associated positively with one group of medium-chain and unsaturated FAMEs often showed negative associations with another group. However, many of these proteins and FAMEs also differed markedly among the four colostrum sources. The hierarchical Mantel analysis separated this source-related covariance from the response to heating. The global correlation was *r* = 0.731 but decreased to 0.297 after within-source centering and to 0.336 when the paired heat-treated-minus-unheated changes were analyzed (Figure 6D). The strong correlation observed across all samples therefore arose mainly from the characteristic protein and fatty-acid composition of each colostrum source, whereas the two molecular layers changed less synchronously after heating.

The RMS response values further illustrate this difference (Figure 6E). Yak showed substantial changes in both the proteome and FAME profile, with RMS values of 0.906 and 0.431, respectively. Donkey and camel had proteomic responses of similar magnitude (0.966 and 0.864) but much smaller FAME responses (0.105 and 0.118). Holstein cow showed the smallest response in both datasets (0.594 and 0.091). This pattern agrees with the single-omic analyses: hundreds of proteins changed in donkey and camel, whereas no individual FAME met the differential criterion in either source; yak, in contrast, showed both the largest FAME response and one of the largest proteomic responses.

The interaction-based correlation analysis gave a similar result at the feature level (Figure 6F). Among the 584 possible combinations of 73 interaction-responsive protein accessions and eight interaction-responsive FAMEs, 85 protein–FAME pairs were significantly correlated across the 12 paired responses. After removal of the source-related component, eight pairs remained significant. The reduction from 85 to eight shows that most of the initial protein–FAME correlations reflected differences among the four colostrum sources, while only a small subset followed the heat-induced changes in parallel.

## 4. Discussion

### 4.1. Interpretation of Heat-Responsive Proteomic Changes

Heat treatment produced substantially more changes in the proteomic profiles than in the FAME profiles, but the direction of these changes must be interpreted in the context of the protein fraction analyzed. DIA proteomics was performed on the centrifuged soluble fraction; therefore, lower abundance after heating does not provide direct evidence that a protein was chemically degraded. Thermal exposure can promote unfolding, intermolecular association, aggregation, and redistribution between soluble and particulate fractions, all of which can reduce the representation of individual proteins in the recoverable soluble fraction [31,32,33]. This distinction is particularly relevant to the predominance of lower-abundance proteins observed in all four sources and to the greater reduction trend observed in the soluble protein fraction of Bactrian camel colostrum. The source-dependent response patterns, including the limited overlap of heat-responsive proteins among the four sources, therefore likely reflect a combination of intrinsic protein thermal sensitivity and matrix-dependent redistribution rather than a single degradation process.

The functional enrichment results further indicate that the molecular consequences of the same heat treatment were not equivalent among sources. Yak showed enrichment of carbohydrate metabolism-related annotations, donkey showed stronger proteasome-, amino-acid-, and glycan-related patterns, and Bactrian camel showed a combination of proteasome, carbohydrate, glycan, and coagulation-related terms. These differences are consistent with reports that whey and MFGM proteins from different dairy species respond differently to common heat-treatment conditions [31,32].

### 4.2. Implications of HTST Processing for Safety and Component Preservation

The 72 °C for 15 s treatment was selected as an industrially relevant high-temperature short-time (HTST) challenge rather than as an optimized pasteurization regime for every colostrum source [12]. In conventional dairy processing, short holding times are used to achieve microbial control while limiting prolonged exposure of heat-sensitive components; however, the effectiveness of this condition cannot be assumed to be identical for colostrum from different species. Recent evaluations of thermal-treatment intensity confirm that increasing heat load progressively affects milk proteins and other bioactive components, whereas short-time pasteurization can retain a greater proportion of heat-sensitive constituents than more intensive treatments [34,35]. Because microbial counts were not measured in the present experiment, our data do not establish pathogen inactivation efficacy for each colostrum source. Instead, they characterize the molecular consequences of a standardized, industrially relevant thermal challenge and provide a basis for future optimization of species-specific safety–quality trade-offs.

### 4.3. Species-Specific Characteristics and Interpretation of Fatty-Acid Responses

The four colostrum sources differ in protein and lipid architecture, which may contribute to their distinct thermal-response patterns. Previous studies have reported distinctive MFGM-associated protein and glycoprotein repertoires in donkey colostrum [36,37], camel colostrum differs markedly from bovine colostrum in major whey-protein composition [38], and comparative studies have demonstrated source-dependent heat responses of whey and MFGM proteins across bovine, camel, yak, and other dairy species [31,32]. These compositional differences provide plausible context for the divergent proteomic responses observed here, although individual functional proteins were not independently quantified and the proposed mechanisms therefore require targeted validation.

The source-level structure observed in Figure 1 further clarifies these differences. Yak and Holstein cow showed the closest proteomic relationship and the fewest pairwise differential proteins (722 before heating), whereas the Holstein–donkey and Holstein–camel comparisons contained 1220 and 1196 differential proteins, respectively. This broader separation of donkey and Bactrian camel from Holstein cow is consistent with comparative evidence showing substantial divergence between donkey and bovine colostrum MFGM proteomes [37] and closer whey-proteome similarity between bovine and yak milk than between bovine and camel milk [39]. These baseline differences in the protein architecture provide a plausible molecular context for why the same 72 °C/15 s treatment produced different sets and magnitudes of heat-responsive proteins among the four sources. Because the animals were sampled from different production environments, however, the pattern should be interpreted as source-associated rather than as a purely genetic species effect.

Fatty-acid profiling was included not because extensive fatty-acid alteration was expected under the relatively mild 72 °C for 15 s condition, but as a complementary molecular layer to determine whether source-associated lipid signatures remained stable or showed selective responses relative to proteins. This expectation was supported by the data: only 14 and six FAMEs changed in yak and Holstein cow, respectively, no significant heat-responsive FAMEs were detected in donkey or Bactrian camel, and the numbers of source-pair differences before and after heating were strongly correlated. Selected fatty acids in bovine colostrum have also shown substantial stability even after longer moderate heating [40], whereas heat can alter milk-fat-globule organization and individual lipid classes without necessarily producing broad changes in total fatty-acid composition [41,42]. Thus, the limited FAME response observed here should be interpreted primarily as preservation of source-associated fatty-acid characteristics under the applied HTST challenge rather than as evidence of extensive lipid remodeling.

From an application perspective, these source-specific protein backgrounds may also contain high-value bioactive targets with potential translational relevance. Donkey milk is known to be particularly rich in lysozyme, an antimicrobial protein with potential applications in functional dairy products and bioactive food ingredients. Bactrian camel milk, meanwhile, contains naturally occurring heavy-chain antibodies, which are of considerable interest for diagnostic, therapeutic, and biotechnological applications because of their distinctive structural properties [43]. However, the present study did not independently quantify the retention or activity of these individual proteins after heating. Therefore, their practical utilization should be further evaluated in terms of recovery efficiency, thermal and functional stability, purification feasibility, scalability, and regulatory requirements. The present omics data may nevertheless provide a useful basis for prioritizing such source-specific high-value components for targeted validation and subsequent application-oriented studies.

### 4.4. Limitations and Future Perspectives

Several limitations should be considered when interpreting the present findings. The relatively small sample size, with three biological replicates per animal source, limits the extent to which the observed patterns can be generalized to the population level; therefore, the differences identified here should be interpreted primarily as source-associated rather than as definitive species-wide characteristics. Although protein content, fat content, and pH were measured, a more comprehensive characterization of the milk matrix, including dry matter, lactose, and ash, would provide additional context for interpreting source-dependent thermal responses. In addition, the four colostrum sources were obtained from commercial farms in different regions and under different feeding and management systems, meaning that the observed source-associated differences may reflect not only intrinsic species-related characteristics but also environmental, dietary, altitude-related, and husbandry factors. Future studies involving larger populations and more harmonized production conditions will therefore be needed to better distinguish these contributions. The present study also evaluated only a single thermal condition, 72 °C for 15 s; comparisons with other processing regimes, including low-temperature long-time and higher-temperature short-time treatments, would help determine whether the observed molecular responses are specific to the applied condition or represent broader processing patterns. Furthermore, all samples were collected on day-3 postpartum, which standardized the lactation stage across animal sources but does not capture the rapid compositional changes occurring throughout early lactation. Finally, the proteomic analysis was restricted to the soluble protein fraction obtained after centrifugation, and future studies incorporating insoluble aggregates, cream-layer proteins, and MFGM-associated proteins would provide a more comprehensive assessment of heat-induced protein redistribution.

## 5. Conclusions

Short-time heat treatment at 72 °C for 15 s induced distinct but source-associated molecular responses in yak, Holstein cow, donkey, and Bactrian camel colostrum. Baseline differences among the four colostrum groups were substantially greater than the alterations induced by heating in both proteomic and fatty-acid profiles, and the overall molecular characteristics of each group were largely preserved after treatment. Heat treatment affected the soluble proteome more extensively than the FAME profile, with heat-responsive proteins detected in all four groups, whereas detectable FAME responses were mainly observed in yak and Holstein cow colostrum. Cross-omic integration demonstrated strong agreement between proteomic and fatty-acid variation at the group level, while heat-induced changes showed weaker concordance between the two molecular layers. Overall, short-time heating caused moderate molecular alterations that depended on the intrinsic characteristics of each colostrum matrix, with broader effects observed in the soluble protein fraction than in fatty-acid composition.

## Figures and Tables

**Table 1:** Protein content, fat content, and pH of colostrum from the four animal sources.

Animal Source	Protein Content (%)	Fat Content (%)	pH
Yak (*Bos grunniens*)	5.61 ± 0.26 ^b^	4.24 ± 0.32 ^a^	6.36 ± 0.09 ^a^
Holstein cow (*Bos taurus*)	5.05 ± 0.24 ^b^	3.97 ± 0.15 ^a^	6.59 ± 0.12 ^a^
Donkey (*Equus asinus*)	2.29 ± 0.27 ^c^	1.10 ± 0.13 ^b^	6.59 ± 0.11 ^a^
Bactrian camel (*Camelus bactrianus*)	6.28 ± 0.11 ^a^	3.96 ± 0.09 ^a^	6.38 ± 0.15 ^a^

Values are expressed as mean ± SD (*n* = 3). Different lowercase superscript letters within the same column indicate significant differences among animal sources (*p* < 0.05), based on one-way ANOVA followed by Tukey’s HSD test.

## Data Availability

The original contributions presented in this study are included in the article/Supplementary Materials. Further inquiries can be directed to the corresponding author.

## Supplementary Materials

The following supporting information can be downloaded at: https://www.mdpi.com/article/10.3390/foods15203609/s1. Supplementary File S1 (peptide.xls): Peptide-level quantitative proteomics data; Supplementary File S2 (protein.xls): Protein-level quantitative proteomics data; Supplementary File S3 (Preprocess Raw814.csv): Fatty acid methyl ester (FAME) profiling data; Supplementary File S4 (Spectronaut-parameter-info.xlsx): Spectronaut software parameters used for proteomic data processing.
